# Evaluation of Human Respiratory Syncytial Virus and Human Parainfluenza Virus Type 3 among Hospitalized Children in Northwest of Iran

**DOI:** 10.1155/2021/2270307

**Published:** 2021-09-22

**Authors:** Zahra Ramezannia, Javid Sadeghi, Shahram Abdoli Oskouie, Mohammad Ahangarzadeh Rezaee, Hossein Bannazadeh Baghi, Arezou Azadi, Mahin Ahangar Oskouee

**Affiliations:** ^1^Department of Microbiology & Virology, Faculty of Medicine, Tabriz University of Medical Sciences, Tabriz, Iran; ^2^Pediatric Health Research Center, Tabriz University of Medical Sciences, Tabriz, Iran; ^3^Infectious and Tropical Diseases Research Center, Immunology Research Center, Tabriz University of Medical Sciences, Tabriz, Iran

## Abstract

**Background:**

Acute respiratory tract infections (ARTIs) are the leading cause of illnesses in children. Human respiratory syncytial virus (HRSV) and human parainfluenza viruses (HPIVs) are among the most common etiologic agents associated with viral respiratory tract infections in children worldwide. Nevertheless, limited information is available on the spread of infections of these two viruses in northwest Iran.

**Objective:**

The purpose of the current study is to evaluate the frequency of RSV and HPIV-3 and clinical features among Iranian children with confirmed respiratory infections between April 2019 and March 2020.

**Methods:**

100 nasopharyngeal swabs were collected from hospitalized patients (under 5 years old) with ARTI from Tabriz Children's Hospital. Detection of respiratory viruses was performed using the nested RT-PCR method.

**Results:**

Respiratory syncytial virus and HPIV-3 were recognized in 18% (18/100) and 2% (2/100) of children, respectively. Ten (55.6%) of the RSV-positive samples were male, while 8 (44.4%) were female. HPIV‐3 was found only among 2 male patients (100%). Most patients (61.1%) with RSV infection were less than 12 months old. Additionally, samples that were positive for HPIV-3 were less than 12 months old. RSV infections had occurred mainly during the winter season.

**Conclusions:**

This study confirms that RSV can be one of the important respiratory pathogens in children in northwestern Iran. However, according to this study, HPIV-3 has a lower prevalence among children in this area than RSV. Therefore, implementing a routine diagnosis for respiratory pathogens can improve the management of respiratory infections in children.

## 1. Introduction

Acute respiratory tract infection (ARTI) is a common reason for hospitalization in children that continues to threaten public health. ARTI is the leading cause of morbidity and mortality among children under 5 in developing or low- and middle-income countries [1, 2]. ARTI is commonly caused by viruses, including human respiratory syncytial virus (HRSV), human parainfluenza virus (HPIV), human bocavirus virus (HBoV), human coronavirus (HCoVs), influenza virus (IFV), and adenovirus (HAdV) [3–6]. Several bacteria also cause ARTI; however, studies show that most ARTIs are associated with viral infections [7]. RSV, which belongs to the family of Paramyxoviridae, is a common cause of acute respiratory tract infections in infants and toddlers that is a cause of bronchiolitis and pneumonia [8]. While RSV infection is usually described as a pediatric illness and a risk factor for asthma in childhood, the immunocompromised and elderly, especially those with previous pulmonary problems, also suffer from this virus [9]. In children under 5 years, annually, RSV can be responsible for 34 million new lower respiratory tract infection (LRTI) episodes and 3.4 million hospital admissions worldwide [1]. Human parainfluenza viruses are another member of the Paramyxoviridae family, one of the leading causes of lower and upper respiratory tract infections in children under five years after RSV. HPIVs have been classified into four serotypes: HPIV-1, -2, -3, and -4. Especially, HPIV-3 is associated with pneumonia and bronchiolitis in children under 1 year, and most children experience it in the first 2 years after birth [10–13]. Nevertheless, little information has been published on the disease burden and epidemiology of HPIV and RSV in the pediatric population of Iran, particularly in the northwest of Iran. This study aimed to determine the frequency of RSV and HPIV-3 in children less than 5 years of age with acute respiratory infection in the northwest of Iran. In addition, we evaluated the clinical manifestations among these hospitalized children in 2019-2020.

## 2. Materials and Methods

### 2.1. Patients and Specimens

This cross-sectional study has been conducted between April 2019 and March 2020. A total of 100 children from birth to 60 months who had shown ARTI symptoms were recruited at the time of hospitalization from Tabriz Children's Hospital. The demographic information, clinical manifestations, and medical records were included in a standardized questionnaire. In addition, nasopharyngeal and throat swabs were collected from each patient by a qualified medical personnel. Specimens were placed in 3 ml of viral transport medium (VTM) and then was delivered to a virology laboratory via a cold chain and stored at −80°C before analysis. Patients with immunosuppression and those with transplants or neoplasia were excluded. Using the nested RT-PCR technique, all specimens were tested for two respiratory viruses, including HPIV-3 and RSV.

### 2.2. Ethics Statement

This study was approved by the Ethics Committee of Tabriz University of Medical Sciences (ethical code: IR.TBZMED.REC.1398.112). Therefore, after obtaining informed written consent from parents, sampling was performed.

### 2.3. RNA Extraction and cDNA Synthesis

According to the manufacturer's instructions, viral RNA was extracted from all samples using the RNX-PLUS kit (Sinaclon, Iran). In brief, 1 ml of RNX-PLUS solution and 200 *μ*l of chloroform were added to the tube containing the sample. Then, the mixture was centrifuged at 12000*g* at 4°C for 15 min. Next, the aqueous phase was transferred to a new tube, and an equal volume of isopropanol was added. The supernatant was discarded after centrifugation, and 1 ml of 75% ethanol was added to the mixture. Afterward, the supernatant was removed, and the sediment was dried at room temperature. The concentration of the RNAs extracted was measured using a spectrophotometer. Following this, complementary DNA (cDNA) synthesis was performed using SinaClon first-strand cDNA synthesis kit according to manufacturer's instructions.

### 2.4. Virus Detection

Primers were synthesized by Pishgam Biotech. Seminested RT-PCR and heminested RT-PCR assays were performed to detect RSV and HPIV-3, respectively (Table 1). This was carried out using master mix PCR (Yektatajhiz, Iran). After cDNA synthesis, in the first PCR to detect RSV, 2 *μ*l DNA template was added to 23 *μ*l of reaction mixture containing 12.5 *μ*l master mix, 8.5 *μ*l distilled water, and 1 *μ*l of each primer (forward 1 and reverse 2) (10 *μ*M) (Table 1). Temperature and time profiles were as follows: 1 cycle at 94°C for 2 min, followed by 40 cycles of 94°C for 45 s, 59.9°C for 30 s, 72°C for 1 min, and final extension at 72°C for 5 min. Then, 2 *μ*l of the first PCR was added to a second PCR (PCR 2) in which the mixture contained 12.5 *μ*l of the master mix, 1 *μ*l of (forward 1) and 2 *μ*l of (reverse 3), and 7.5 *μ*l of distilled water in a final volume of 25 *μ*l. Temperature and time profiles at this step were as follows: 1 cycle at 94°C for 2 min, followed by 40 cycles of denaturing, annealing, and extension (45 s at 94°C, 30 s at 54.5°C, and 1 min at 72°C), and final extension 5 min at 72°C.

**Figure 1:**
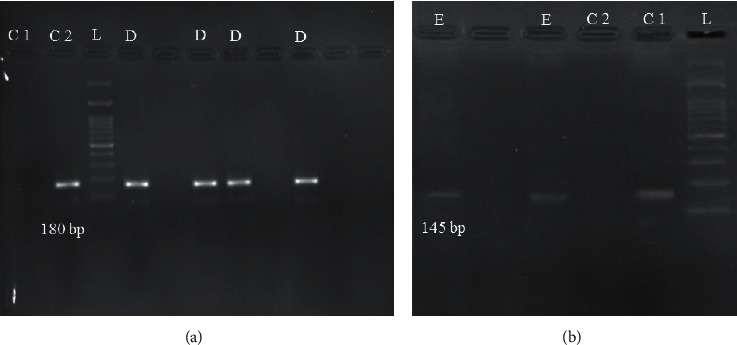
Agarose gel electrophoresis of the nested RT-PCR of RSV (a) and HPIV-3 (b). In (a), C1: negative control; C2: positive control; L: 100-bp DNA ladder; D: RSV-positive samples (180-bp). In (b), C1: positive control; C2: negative control; L: 100-bp ladder; E: HPIV-3-positive samples (145-bp).

The steps and PCR reaction mixture for detection of HPIV-3 were the same as RSV, but with specific HPIV-3 primers (Table 1). Temperature and time profiles were as follows: for the first PCR, 1 cycle at 94°C for 2 min, followed by 40 cycles of 94°C for 45 s, 56.9°C for 30 s, 72°C for 1 min, and final extension at 72°C for 5 min; and in the second PCR, 1 cycle at 94°C for 2 min, followed by 40 cycles of 94°C for 45 s, 52.6°C for 30 s, 72°C for 1 min, and final extension at 72°C for 5 min. The negative (distilled water) and positive (RSV and HPIV-3 known samples) controls were placed in each run (Figure 1).

### 2.5. Statistical Analysis

Frequency and percentage were used to display qualitative data (sex, age groups, clinical manifestations of patients, and type of virus isolates). The Chi-square test was used to compare the qualitative data between types of virus isolates. The data were analyzed using SPSS software version 21. Statistical significance level was considered below 0.05.

## 3. Results

### 3.1. Patient Characteristics

Of 100 enrolled children (under 5 years old), 44 were girls and 56 were boys. No significant difference was observed in gender. The age distribution of the study population was as follows: less than 12 months (53%), 13–36 months (33%), and 37–60 months (14%). RSV and HPIV-3 tests were positive in 18 (18%) and 2 (2%) patients with acute respiratory tract infection, respectively. There was no statistically significant association between RSV infection and HPIV-3 infection with patients' age and gender (*P* > 0.05) (Table 2).

### 3.2. Seasonal Distribution

Among the samples obtained from April 2019 to March 2020, cases of RSV were observed during the study period. From December, an increase of positive cases was observed, particularly in January and February, and a decrease during spring and summer (*P*=0.002). Additionally, HPIV-3-positive samples were detected in the spring.

### 3.3. Respiratory Pathogens and Clinical Characteristics

Clinical features for these two viral infections were examined. Symptoms recorded in patients included fever, wheezing, cough, vomiting, diarrhea, pneumonia, runny nose, cyanosis, tachypnea, and dyspnea. Common symptoms in children with RSV infection included wheezing (66.7%), cough (61.1%), fever (55.6%), vomiting and pneumonia (33.3%), and runny nose (27.8%). In addition, children recognized with RSV had a higher proportion of tachypnea (*P*=0.005) than children that were not. Furthermore, tachypnea was predominant in boys with RSV infection (*P*=0.043) (Table 3).

## 4. Discussion

This study indicated that RSV and HPIV-3 are among the important respiratory pathogens associated with acute respiratory infections in young children in northwest Iran. In the present study, the frequency of RSV and HPIV-3 in children under 5 years of age was 18% and 2%, respectively. Overall, there is limited evidence on the epidemiological burden of RSV and HPIV-3 in the northwest of Iran in the published literature. According to the results of a meta-analysis from 1996 to 2013, the prevalence of RSV infection in Iran was reported to be 18.7% [15]. One of the studies that investigated the prevalence of RSV in northwestern Iran was conducted by Jedari et al. in 2002; they evaluated RSV infection in children under 5 years of age with lower respiratory tract infection by immunofluorescence assay and reported that RSV was the cause of 24.6% of the cases [16]. Pourakbari et al., in 2014, found that, among 232 children with acute respiratory infections, 17.2% were RSV positive in Tehran, Iran [17]. In another study from Tehran, the prevalence of RSV among children between 2008 and 2009 was 16.8% [14]. According to the mentioned results above, the results of our study are similar to the prevalence of RSV in other studies led in Iran. However, additional studies have been conducted in southern Iran, which shows a higher percentage of the prevalence of RSV than our study. For instance, one study found that 30% of children with ARI were positive for RSV in Shiraz in 2015 [18]. Furthermore, according to a systematic review study from 2001 to 2019 in the Middle-East and North Africa, the prevalence of RSV is generally 24.4%, higher than our study's results [19]. Similarly, a study from India conducted between August 2011 till 2013 reported a 24.5% prevalence of RSV among children under 5 years of age [20]. In this study, RSV was mostly found in male infants. Likewise, most positive cases were less than one year old, similar to other studies [14, 21]. There was no difference in clinical symptoms such as wheezing, fever, cough, vomiting, diarrhea, pneumonia, runny nose, and cyanosis in RSV-positive patients compared to RSV-negative patients and only a significant difference between the two groups in tachypnea. However, tachypnea was more common in RSV-positive patients. Like our study, Lu et al. in China reported that tachypnea was more frequent in RSV-positive infants than in RSV-negative infants [22]. Meanwhile, wheezing, cough, and fever were the common clinical signs and symptoms in these children. Moattari et al. reported that cough, wheezing, and bronchiolitis were more common in RSV-positive patients; however, similar to our study, they found no difference in other clinical symptoms between the two groups [18]. Since there are no other studies reported on the prevalence of parainfluenza virus 3 or other types in the northwest of Iran, our study results gain significance. The human parainfluenza virus 3, like most RSV-positive samples, was found in infants less than 12 months of age, and also, HPIV-3-positive samples were found in the spring. The prevalence of HPIV-3 in our study was almost close to other parts of Iran, such as Tehran. In a study conducted in 2010, the HPIV-3 rate of 4.4%, and in another study in 2014, a prevalence of 8.4% for HPIV-1–4 were reported by Shatizadeh et al. [14, 23]. Al-Ayed et al. found a 1.8% HPIV-3 frequency in children under 5 years with ARTI diagnosis between October 2012 and July 2013 in Saudi Arabia [24]. Variously, in other Asian countries such as Bangladesh, the prevalence of HPIV-3 was reported to be 10.5% among children between August 2014 and July 2015 [25]. In addition, a recent study reported that the prevalence of HPIV in children under 5 years of age in most Middle-East countries was more than 15%, but did not specifically report the prevalence of HPIV-3 [26]. In the present study, the RSV seasonal distribution is defined by the fall and winter peaks. Our study shows a lower prevalence of RSV in spring, which, according to previous studies in Iran, have been diagnosed with RSV infection during the cold season from November to March [15]. In other parts of the world, the seasonal distribution of RSV can vary depending on geographical location, high precipitation rate, and cold temperature. But in general, strong seasonal distribution with high prevalence during the winter is observed in most places [19]. Further epidemiological studies are needed to evaluate the seasonal patterns of RSV and HPIV infections in Iran. There are some limitations in this study, including the relatively small sample size and the lack of some clinical information that can be mentioned. Also, recognizing other viral pathogens, other HPIV types, and RSV strain identification and virus genotyping has not been investigated in this study. Several studies have reported a high rate of RSV coinfection with other respiratory viruses and bacterial pathogens. Hence, in analyzing the burden of illness associated with RSV infection, coinfections are a major confounder that cannot be ignored [27]. Some factors allow us to attain a more accurate assessment of the true burden of RSV and other respiratory viruses in the northwest of Iran, such as further studies on outpatients with acute respiratory infections, annual monitoring of the prevalence of respiratory viruses, and considering children with special conditions such as premature infants. This information will be important for understanding viral epidemics.

## 5. Conclusion

According to our findings, RSV can be one of the important respiratory pathogens in children in northwestern Iran. Also, HPIV-3 had a low prevalence among children in this area than RSV. Consequently, implementing a routine diagnosis for respiratory pathogens can improve the management of respiratory infections in children and help combat ARTI.

## Figures and Tables

**Table 1:** Sequences of oligonucleotides used for detection of RSV and HPIV-3.

Primers name	Target gene	Sequence (5′ to 3′)	Product size (bp)	Reference
Seminested RT-PCR for RSV	N	Forward (1): GGAACAAGTTGTTGAGGTTTATGA ATATGC	210	[14]
		Reverse (2): TTCTGCTGTCAAGTCTAGTACACT GTAGT		
		Reverse (3): TTCTGCTGTCAAGTCTAGTACACTGTA GT	180	
Heminested RT-PCR for HPIV-3	HN	Forward (1): CTCGACGTTGTCAGGATATAG	189	
		Reverse (2): CTTTGGGACTTGAACACAGTT		
		Reverse (3): GCTAGAGAACATGACTTCC	145	

*Abbreviations*. N: nucleocapsid; HN: hemagglutinin-neuraminidase.

**Table 2:** Characteristics of the study population with ARTI disease (*n* = 100).

Variable	Total (*N* = 100)	RSV			HPIV-3		
		Positive (*N* = 18)	Negative (*N* = 82)	*P* value	Positive (*N* = 2)	Negative (*N* = 98)	*P* value
Gender				0.967			0.205
Male	56 (56.0%)	10 (17.9%)	46 (82.1%)		2 (3.6%)	54 (96.4%)	
Female	44 (44.0%)	8 (18.2%)	36 (81.8%)		0 (0.0%)	44 (100%)	
Age groups (month)				0.746			0.405
≤12	53 (53.0%)	11 (20.8%)	42 (79.2%)		2 (3.8%)	51 (96.2%)	
13–36	33 (33.0%)	5 (15.2%)	28 (84.8%)		0 (0.0%)	33 (100%)	
37–60	14 (14.0%)	2 (14.3%)	12 (85.7%)		0 (0.0%)	14 (100%)	

*Abbreviations.* ARTI: acute respiratory tract infection; RSV: respiratory syncytial virus; HPIV-3: human parainfluenza virus 3.

**Table 3:** Association of symptoms with respiratory syncytial virus infection and parainfluenza 3.

Clinical manifestations of patients	RSV			HPIV		
	Positive	Negative	*P* value	Positive	Negative	*P* value
Wheezing			0.003			0.28
Yes	12 (33.3%)	24 (66.7%)		0 (0%)	36 (100%)	
No	6 (9.4%)	58 (90.6%)		2 (3.1%)	62 (96.9%)	
Fever			0.17			0.33
Yes	10 (14.5%)	59 (85.5%)		2 (2.9%)	67 (97.1%)	
No	8 (25.5%)	23 (74.2%)		0 (0.0%)	31 (100%)	
Vomiting			0.46			0.79
Yes	6 (14.6%)	35 (85.4%)		1 (2.4%)	40 (97.6%)	
No	12 (20.3%)	47 (97.7%)		1 (1.7%)	58 (98.3%)	
Diarrhea			0.12			0.23
Yes	1 (5.6%)	17 (94.4%)		1 (5.6%)	17 (94.4%)	
No	17 (20.7%)	65 (79.3%)		1 (1.2%)	81 (98.8%)	
Pneumonia			0.08			0.25
Yes	6 (31.6%)	13 (68.4%)		1 (5.3%)	18 (94.7%)	
No	12 (14.8%)	69 (85.2%)		1 (1.2%)	80 (98.8%)	
Cough			0.21			0.39
Yes	11 (15.1%)	62 (84.9%)		2 (2.7%)	72 (97.3%)	
No	7 (25.9%)	20 (74.1%)		0 (0%)	26 (100%)	
Runny nose			0.10			0.88
Yes	5 (11.1%)	40 (88.9%)		1 (2.2%)	44 (97.8%)	
No	13 (23.6%)	42 (76.4%)		1 (1.8%)	54 (98.2%)	
Cyanosis			0.63			0.83
Yes	0 (0%)	1 (100%)		0 (0%)	2 (100%)	
No	18 (18.2%)	81 (81.8%)		2 (2%)	96 (98%)	
Tachypnea			0.005			0.69
Yes	4 (57.1%)	3 (42.9%)		0 (0%)	7 (100%)	
No	14 (15.1%)	79 (84.9%)		2 (2.2%)	91 (97.8%)	
Shortness of breath			0.85			0.016
Yes	5 (19.2%)	21 (80.8%)		2 (7.7%)	24 (92.3%)	
No	14 (17.6%)	61 (82.4%)		0 (0%)	74 (100%)	

*Abbreviations.* RSV: respiratory syncytial virus; HPIV-3: human parainfluenza virus 3.

## Data Availability

The data used to support the findings of this study are included within the article.
